# Effect of Diet on the Gut Microbiota: Rethinking Intervention Duration

**DOI:** 10.3390/nu11122862

**Published:** 2019-11-22

**Authors:** Emily R Leeming, Abigail J Johnson, Tim D Spector, Caroline I Le Roy

**Affiliations:** 1 Department of Twin Research & Genetic Epidemiology, King’s College London, St Thomas’ Campus, Lambeth Palace Road, London SE1 7EH, UK; emily.leeming@kcl.ac.uk (E.R.L.); tim.spector@kcl.ac.uk (T.D.S.); 2 BioTechnology Institute, University of Minnesota, Saint Paul, MN 55108, USA; cole0463@umn.edu

**Keywords:** gut microbiota, diet, nutrition, dietary intervention, duration, health

## Abstract

The human gut is inhabited by trillions of microorganisms composing a dynamic ecosystem implicated in health and disease. The composition of the gut microbiota is unique to each individual and tends to remain relatively stable throughout life, yet daily transient fluctuations are observed. Diet is a key modifiable factor influencing the composition of the gut microbiota, indicating the potential for therapeutic dietary strategies to manipulate microbial diversity, composition, and stability. While diet can induce a shift in the gut microbiota, these changes appear to be temporary. Whether prolonged dietary changes can induce permanent alterations in the gut microbiota is unknown, mainly due to a lack of long-term human dietary interventions, or long-term follow-ups of short-term dietary interventions. It is possible that habitual diets have a greater influence on the gut microbiota than acute dietary strategies. This review presents the current knowledge around the response of the gut microbiota to short-term and long-term dietary interventions and identifies major factors that contribute to microbiota response to diet. Overall, further research on long-term diets that include health and microbiome measures is required before clinical recommendations can be made for dietary modulation of the gut microbiota for health.

## 1. Introduction

The gut microbiota is a complex ecosystem predominantly found in the colon. Compositional or functional changes within the gut microbiota have been shown to contribute to both health and disease including immune, metabolic and neuro-behavioural traits [1,2]. Gut microbiota composition can be highly variable between individuals, though some key bacterial species are typically present in most. Diet is thought to explain over 50% of these microbial structural variations in mice and 20% in humans signalling the potential for dietary strategies in disease management through gut microbiota modulation [3,4]. Short-term, dramatic dietary interventions have demonstrated the ability to alter microbiota diversity quickly in humans [5]. However, these alterations are transient and do not persist for more than a few days [5]. Even after extensive dietary changes, an individual’s microbiota maintains its unique personalised composition [6] suggesting that the forces controlling ecological homeostasis extend beyond diet. However, when viewed cross-sectionally across populations, dietary patterns also correspond with microbial composition [6]. This suggests that long-term dietary patterns and habitual intake play a role in shaping each individual’s stable microbiota profile. What remains unclear, however, is an understanding of how long a dietary intervention would need to be to permanently alter the ecological homeostasis of the microbiota community, such that after removal of the intervention a different state of ecological homeostasis persists.

Besides diet, the gut microbiota is shaped by a combination of extrinsic (e.g., lifestyle and medication) and intrinsic (e.g., host genetics, immune and metabolic regulations) factors. It is generally acknowledged that the extrinsic factors elicit the predominant effect, with diet the most widely studied [4]. Genetics only perform a limited role in shaping the gut microbiota with an average level of 8.8% according to a 2016 data analysis of 1126 twins [7]. In turn, the gut microbiota composition is highly individualised to the host and shaped across a lifetime with this unique make up of bacterial taxa commencing at birth [8]. 

Key influences on the neonatal microbiota include the mode of infant delivery [9], method of infant feeding [10] and medication usage (in particular antibiotics) [8]. The gut microbiota undergoes dramatic changes soon after birth with lactation followed by a secondary shift on the introduction of solid foods [11]. During this time, the infant is subject to low bacterial diversity and a high rate of microbial flux until around 2–3 years of age [12]. This window is a period of critical development of the gut microbiota, with disruptions linked to a higher risk of autoimmune diseases and metabolic disturbances in later life [11,13]. Vatanen et al. observed that the composition and function of the microbiota in infants, and not simply overall microbial diversity, may be equally important to health parameters [13]. For example, a heightened quantity of lipopolysaccharide (LPS) producing subtypes during this time can increase immune activation and possibly mediate autoimmune diseases in later life, acknowledged to be on the rise in the western world [14]. After around 3 years, the gut microbiota stabilises retaining relative proportions of taxa with adaptations to composition harder to impose [15]. In this way, the first years of seeding and establishing the core gut microbial profile may play a fundamental role in host health in adulthood. After three years of age environmental factors such as diet and antibiotics, but also disruption of host metabolic and immune homeostasis, can still influence or disturb microbial composition. The microbiota’s resilience to perturbations depends on the responsive capabilities of the inherent core taxa to return to its normal state and function [15]. While this resilience to change can be inherently protective from loss of keystone taxa, it may also inhibit the amelioration of a disrupted microbiota.

Permanent rather than transient changes to the core gut microbiota are likely required for long-term impact on health outcomes [16]. Durable bacterial implantation and proliferation may require continuous substrate availability. With habitual diet thought to play an integral role in shaping the gut microbial environment, diet–microbe interventions must consider the capabilities of an individual to make sustainable dietary changes. Thus, permanent alteration of the diet may induce new species and proliferate others, increasing the diversity and richness of beneficial taxa. In this way, a new state of ecological homeostasis of the gut microbiota may be achieved, mediated by diet with beneficial implications for host health. One of the greatest challenges to understanding the relationship between diet, the microbiota and health, however, is to decipher the high variability in individual responses to food intake. These wide variations contribute to many conflicting outcomes in this area of research with failures to find diet-specific effects [16]. The homogenisation of study outcomes is imperative in improving our understanding of dietary interventions on the microbiota for enhanced health.

This review describes the current state of knowledge regarding the duration of time required for a dietary intervention to impact the gut microbial signature. A durable impact on the gut microbiota may be considered as one that induces a new state of ecological homeostasis of the gut microbiota. The implications for future research investigating diet and the gut microbiome will be highlighted. For the sake of this literature review, short-term dietary interventions were considered to be those investigating the immediate impact to a study duration of a number of months, with long-term studies considered to be predominantly epidemiological in nature or of an extended time period (≥6 months).

## 2. Acute Dietary Exposure and the Gut Microbiome

### 2.1. The Gut Microbiota Responds Rapidly to Dietary Changes

While the core bacterial taxa are resilient to most temporary outside influences, the gut microbial community as a whole displays a high inter-individual day-to-day variability [2]. Gut microbes are extensively and regularly purged and have the ability to double in number within one hour [17]. Within 24–48 h of a dietary intervention rapid changes are thought to be made to the microbial composition on a species and family level (but not phyla) [17]. Likewise, mouse models have indicated that manipulation of macronutrient intake can consistently shift the composition of the gut microbiota within the span of a day [5,6,18,19]. This variability is explained only in part by the diet composition itself, with a number of intrinsic and extrinsic factors thought to contribute such as circadian rhythm and feeding behaviours [20,21].

Although the gut microbiota is not exposed to the light and dark cycle associated with the circadian rhythm, its composition and function are thought to still be affected by this cyclical ebb and flow [22]. In humans, at least 10% of Operational Taxonomic Units (OTUs) oscillate due to the circadian rhythm [22]. The microbiota fluctuates based on nutrient availability and the level of host-derived auto-antibodies and peptides, both of which are associated with circadian rhythm oscillations [20,22,23]. The microbiota is thought to programme these synchronised diurnal oscillations by rhythmic histone acetylation through epithelial histone deacetylase 3 (HDAC3). HDAC3 integrates microbial and circadian cues, through which metabolic gene expression and nutrient intake are affected. This interaction regulates intestinal lipid uptake, with disruption potentially promoting diet-induced obesity [24]. Jet-lag, an acknowledged disrupter of the body’s internal clock and eating patterns, has been suggested to lead to changes in microbial composition in an exploratory study in two humans and mice that promoted glucose intolerance and obesity [22]. Likewise, disrupted sleep patterns, often common in shift workers, has been found to alter the gut microbiota, increase dietary intake and promote an inflammatory response that can incite metabolic stress [25].

The feeding regimen itself has a powerful training effect on peripheral oscillators such as the liver and intestine [26]. It is possible therefore that manipulation of feeding time, including time and duration of consumption and frequency, may influence the gut microbial composition and function and possibly host health [21,27]. In humans, Kaczmarek et al. observed that several bacteria were related to the time of eating [27]. Likewise, in mouse models Thaiss et al. displayed that a rhythmic food intake not only leads to 15% of commensal bacterial taxonomic units fluctuating throughout the course of a day, but also increased microbial abundance [22]. The impact of meal timing in humans on the gut and oral microbiotas was explored in a 2018 randomised crossover study by Collado et al. (*n* = 10). They found that the timing of a meal can affect the diurnal rhythms of the salivary microbial profile with eating the main meal late (at 17:30 as opposed to 14:30) shown to increase salivary taxa generally considered to be pro-inflammatory; affecting body weight, cortisol rhythm, basal metabolic rate, glucose tolerance and body temperature [28]. With the gut microbiota able to fluctuate in as short a time span as an hour [17], poses the question of whether hunger related to delayed feeding could potentially manipulate the composition of the gut microbiota. However, no significant effect of eating early or late was observed on the overall faecal microbial composition in the Collado et al. study [28]. In a mouse model, a number of bacteria and bacterial metabolites have been shown to be involved in regulation of hunger and satiety, with their production dependent on bacterial growth cycles [29]. Fundamental characteristics of the effect of fasting or time-restricted feeding on the gut microbiota are still unknown with a limited number of observational studies of religious fasting and some modest experimental studies, most with fewer than 50 participants [30].

Due to our co-evolution with our gut ecology [31], the gut microbiota’s ability to rapidly respond to dietary changes may be reflective of our volatile hunter-gatherer dietary intake that was based on necessity for dietary flexibility with periods of feast and famine [18]. One longitudinal study involving daily gut microbiota investigations of two individuals over the course of a year found that changes in fibre intake are positively correlated with a change in abundance of 15% of the microbial community the following day [5]. These relatively rapid changes to the gut microbiota could be a ‘shock reaction’ to an influx of incoming nutrients, possibly causing a transient disruption of microbial composition [32]. The ability of the gut microbiota to cope with this stress is part of the inherent plastic nature of the normal microbiota. In this way, the gut microbiota is able to adapt and adopt a new beneficial or detrimental state when faced with a continuous perturbation [33]. However, the duration of any intervention required to illicit a permanent change to the core microbial profile is still unknown, with most producing only transient fluctuations within the community [18].

### 2.2. Effect of Short-Term Dietary Interventions on the Gut Microbiota

Significant microbial changes have been noted amongst only a limited number of bacterial taxa during short periods of dietary interventions [22]. In humans, there are rapid but transient changes in the gut microbiota in response to a dietary intervention particularly in the first 24-h period. However, enterotypes, the distinct bacterial groupings of the core microbial profile, are thought to remain stable throughout an intervention [6]. A number of dietary studies have detailed no significant effect of diet on the microbiota, possibly as a result of interpersonal variability in enterotype composition though this may be overwhelmed by a suitably extreme diet [34].

Transient changes to the gut microbiota composition with extreme diets have been noted in several studies [12]. David et al. observed the effect of two dietary regimens in a cross-over design, one almost exclusively plant-based and the other almost exclusively animal-based. Each diet was consumed ad libitum by 10 subjects for 5 consecutive days. Both diets shifted gut microbiota composition [35], with the animal-based diet displaying significantly decreased levels of carbohydrate fermentation faecal metabolites and increased amino acid fermentation faecal metabolites within 24 h, in comparison to baseline and the plant-based diet [18]. However, the microbiota of the participants returned to baseline within 3-day post-dietary intervention [18].

The gut microbiota can also rapidly respond to altered macronutrient levels and novel food components. A controlled feeding experiment by Wu et al. investigated the effect of high fat/low fibre and low fat/high fibre diets on 10 randomised study participants. The faecal microbiota of all 10 individuals displayed dramatic albeit temporary shifts, though inter-subject variability remained high even after periods of identical dietary intake [6]. Fibre content, amount and type appear to be pivotal determinants of microbiota composition [36]. A 2018 systematic review and meta-analysis observed the effect of fibre on the gut microbiota from 64 studies. Dietary fibre interventions, particularly fructans and galactooligosaccharides (GOS), were found to increase faecal abundance of Bifidobacterium and Lactobacillus species but did not affect alpha-diversity [37]. In terms of fibre food sources, Johnson et al. found statistically significant impact of fruit and grain fibre on gut microbiota composition [35]. Johnson et al. developed and applied new multivariate methods for modelling dietary intake for their 17-day longitudinal study in which they collected daily faecal microbiota samples and dietary data which was imputed into a whole-food phenetic hierarchical structure [35]. They found that microbial composition was more strongly related with food choices rather than the conventional nutrient profile typically used in nutrition research, though highly personalised responses were displayed.

Collectively, studies show that alterations made in diet can have a significant and meaningful effect on the gut microbiota, primarily influenced by fibre from fruits, vegetables and other plant foods. However, in short-term interventions, faecal analyses are typically taken during the study period and not afterwards, as displayed in Table 1. As a result, this hampers our understanding of the duration of an association between the gut microbiota and diet. Cross-over study designs typically contain wash-out periods between diet intervention arms where a microbiota sample may be taken at the end of one study arm, and after a period of days or weeks, a microbiota sample is taken again at the beginning of the next. These studies provide a way to indirectly observe if an intervention has a lasting effect on the gut microbiota beyond the intervention stage. For example, a randomised double-blind cross-over study by Liu et al. observed that consumption of fructooligosaccharide (FOS) and GOS for 14 days increased Bifidobacterium and reduced butyrate-producing bacteria in 35 healthy adults. However, after a 28-day washout period, the gut microbiota was shown to recover to its pre-intervention baseline state displaying that without continued consumption of these prebiotic fibres, the noted microbial changes are lost within the 28-day wash-out period [38]. Burton et al., also found that there was an absence of the probiotic bacterial strains related to the two-week consumption of probiotic yoghurt after a three week wash-out period (*n* = 14) [39]. In a study by Kellingray et al., increased consumption of Brassica was associated with reduced relative abundance of sulphate-producing bacteria and members of Rikenellaceae, Ruminococcaceae, Mogibacteriaceae, and Clostridiales [40]. Though, they observed little evidence of carry-over effects of high-Brassica diet after a two-week wash-out periods. The transient nature of these diet-induced microbial changes disappearing shortly after cessation of a dietary initiative suggest that continual intake of the nutritional substrate may be required. This suggests the importance of sustainable changes to the habitual diet for maintenance of this dietary effect on gut microbial composition.

The magnitude of the effect on the gut microbiota in short-term dietary interventions is thought to be relatively modest in relation to the inter-individual variability of the microbial profile [41]. Thus, short-term interventions may be of too limited a duration to have a long-standing impact on gut microbiota composition. With daily dietary intake providing continuous provision of substrates to the gut microbiota, thereby shaping the gut microbial environment, this may be expected. Extreme dietary shifts, however, are thought to illicit a more pronounced effect [41]. To establish therapeutic dietetic strategies on the microbiota, improved understanding of the immediate and particularly the ongoing relationship between diet and the gut microbiota is required. Other gut-directed dietary interventions tested on a short timespan are related to the use of pre and probiotics.

### 2.3. The Impact of Probiotics on Microbial Communities Is Individualised and Transient

Probiotics have been defined as “live microorganisms which, when administered in adequate amounts, confer a health benefit on the host” [42]. Many probiotic bacteria are traditionally used in the fermentation of food but are now predominantly ingested by the public as supplement-like probiotic products that contain live bacteria. After consumption probiotics have the capacity to colonise and proliferate within the gastrointestinal tract thereby influencing the gut ecosystem. There is increasing popular interest in the potential benefits of probiotics. However, clinical evidence may sometimes be contradictory, mostly as a result of low study power and potential variability in the strains used between two studies. While there is limited evidence around many probiotics, some systematic reviews and meta-analyses have supported that specific strains may be effective in certain areas [43,44,45,46]. Findings have indicated the benefit of probiotics in aiding the treatment of infectious- and antibiotic-associated diarrhoea, insulin resistance in diabetes, and remission and maintenance of inflammatory bowel disease, amongst others [44,45,46,47]. However, probiotic outcomes can be unpredictable and individualised. The ability of a probiotic strain to establish itself within the gut microbial community can be highly variable; the strain may need to compete against the host’s resident microbes for substrates in tandem with resisting antimicrobial peptides, thus establishing an ecological niche [48]. Zmora et al. displayed that colonisation in the gut microbiota by probiotics occurs in highly individualised patterns, with engraftment occurring in some and not in others [49]. Maldonado-Gómez et al. demonstrated that personalised engraftment of a probiotic was dependent on the availability of an open ecological niche [50]. Rejection is highly prevalent in healthy individuals with little evidence that probiotics have a substantive impact on the gut microbial profile besides a transient increase that rarely persists [51]. In fact, a 2016 systematic review by Kristensen et al. found that probiotics have no impact on alpha-diversity, evenness and richness in the faecal microbiota across several studies on healthy individuals, bar one which noted an effect on beta-diversity [52]. This may be due to the nature of a healthy and diverse gut ecosystem that either competitively impedes the engraftment of a new strain or is already found to be present within the community. However, this begs the question of whether the enduring establishment of a probiotic within the gut microbiota is required to elicit a beneficial effect. While probiotics may be transient, they have the capacity to alter the composition of the gut microbiota, in turn influencing the production of beneficial fermentation-derived metabolites [53]. Probiotic studies tend to measure clinical outcomes rather than colonisation of the probiotic strain, with significant results suggesting that colonisation isn’t necessarily required to reap health benefits from probiotic ingestion [51]. A study by Meance et al. described the long-lasting effects of the probiotic strain Bifidobacterium animalis DN-173 010 (BM) on transit time after two-week consumption by 200 elderly individuals. On conclusion of the study, the reduced length of transit time observed only returned to baseline at 6-week follow-up for those with a medium transit time (40–50 h), and at 4-week follow-up for those with slow transit times (>50 h) who had been consuming 150 g of BM per day [54].

### 2.4. Prebiotics Induce Changes in Microbial Composition and Metabolite Production

Plant-based foods such as fruit, vegetables, legumes, grains and nuts contain dietary fibre. While fibre as a whole is generally accepted to be beneficial to gastrointestinal health, specific dietary fibre types including inulin, FOS and GOS are also considered to be prebiotic; defined as “a substrate that is selectively used by host microorganisms conferring a health benefit” [55,56]. The definition of prebiotics goes beyond these traditional compounds to include any food component that reaches the large intestine and elicits a selective effect on microbial growth to confer a health benefit. These compounds are resistant to gastric acidity and hydrolysis by digestive enzymes, bypassing absorption in the upper intestine to the colon where they are metabolised by the microbiota. Biotransformation of these food components often results in the production of short-chain fatty acids (SCFAs), including acetate, propionate and butyrate [57].

Prebiotic consumption has been associated with growth of Bifidobacterium, Lactobacillus and lactic acid bacteria. Conversely, fibres not classified as prebiotics, do not appear to affect the abundance of Bifidobacterium or Lactobacillus [37]. Gurry et al. randomised 60 individuals into one of several 6-day dietary arms of a highly controlled feeding study investigating the effect of a number of prebiotics and other nutrients on the gut microbiota [58]. They demonstrated strong and predictable responses of specific microbes to the prebiotic arms (pectin and inulin) but not the other non-prebiotic micronutrient arms, consistently across all participants. The response to cellulose, however, was seen to be highly variable between individuals [58]. Health outcomes have also been associated with prebiotic intake. For example, a study by Dewulf et al. investigating the effect of inulin-type fructan on women with obesity for three months, found a shift in microbial profile and altered fat metabolism. Consequentially, a reduction in fat mass, serum LPS and some metabolites such as phosphatidylcholine, lactate and hippurate were displayed [59]. Increasingly, our understanding of prebiotics suggest that they not only have a positive impact on gut microbial composition, but also health outcomes through the production of beneficial metabolites. Many of the changes appear to be transient and suggest that habitual consumption is required for continued beneficial health outcomes related to prebiotic intake.

Food sources of fibre, such as whole grains, have been suggested to have a prebiotic-like effect on the gut microbiota [60]. Roager et al. investigated the effect of two 8-week diet intervention periods of either a whole grain diet, or a refined grain diet, on the microbiota and health markers of 60 adults at risk of developing metabolic syndrome in a randomised, controlled, cross-over design study [61]. They found that the higher fibre whole grain intervention had no significant impact on the gut microbial composition, diversity or functional potential in comparison to a refined grain intake. However, there were marked improvements in inflammatory biomarkers even after adjusting for weight loss [61]. Vuholm et al. also investigated the effect of whole grain wheat and rye vs refined wheat, with improvements displayed in health parameters but no significant effect between whole grains and refined grains on the gut microbiota. A randomised, controlled, parallel designed study by Vanegas et al., however, displayed a modest effect on the microbiota at the family and genera level between the participants consuming whole grain (*n* = 41) compared to those consuming refined grain (*n* = 40) [62].

## 3. Long-Term diet and the Gut Microbiome 

### 3.1. Evidence for Long Lasting Effect of Diet on the Gut Microbiome

**Figure 1:**
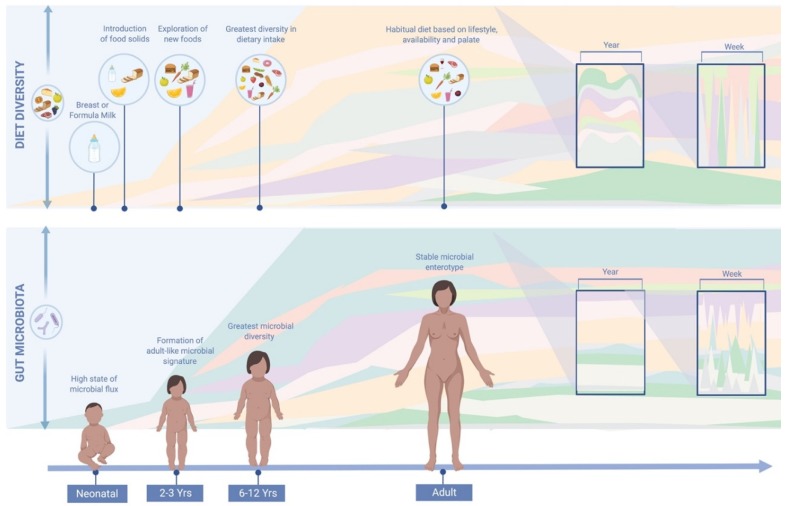
Comparison of diet and gut microbiota variations throughout life. Habitual diet plays a role in shaping the gut microbial environment, and hence, microbial composition. Dietary diversity has been associated with microbial diversity [78]. Throughout the year, the human diet tends to display a cyclical seasonal pattern due to seasonal availability and dietary preferences. Large day to day variations in diet are not reflected in the gut microbiota, suggesting that overall dietary habits have a greater impact on gut microbial composition [35]. This image was generated using BioRender Software (http://www.biorender.com/).

Accumulating evidence has suggested that long-term diet is the primary driver of gut microbiota composition, as depicted in Figure 1. However, most of these observations were made on cross-sectional studies. As described in the introduction it is the first three years of life that diet, together with other factors, appears to have the greatest impact on microbial ecology [11]. By three years of age, a more stable and adult-like microbial signature is thought to have been established with greater resistance to perturbations. The gut microbiota, however, may undergo a more prolonged development than previously suspected, with other evidence to indicate that it continues to develop past early childhood [82,83]. The microbial biodiversity of six to twelve year olds, for example, has been shown to be greater than those of healthy adults [84,85]. In a cross-sectional study by Hollister et al., pre-adolescent children had greater dietary diversity than adults with a higher aptitude for testing and exploring new foods. In adulthood, a habitual dietary pattern tends to be established based on lifestyle, palate and accessibility, with a lower propensity for trialling new food types [84,85]. Despite a tendency for microbial stability in adulthood, nutrient quality and quantity may still impact and derail the gut microbiota [12,18]. Habitual diet provides a consistent source of dietary substrates to the microbiota, creating an environment that continuously shapes microbial ecology [34]. Habitual diet has been shown to be associated with a distinct compositional enterotype, while short-term dietary intake has not [6]. It is these core gut microbial groups that are thought to be resilient to perturbations and which have been associated with a number of microbiome-disease associations along with differential metabolic responses to medication [86]. Inter-individual differences in enterotype composition are increasingly thought to explain the variation in response to a dietary intervention or perturbation [86].

The effect of seasonality on both diet and the microbiome has only recently been explored in the current literature. Free ranging animal models have suggested that seasonality may affect the function and composition of the gut microbiota [87,88] in response to a seasonal change in diet. In 2017, researchers collected 350 faecal samples from the Tanzanian Hadza tribe over the span of a year [76]. The microbiota of these hunter-gathers were shown to reflect the seasonality of their diet, with food type and availability affected by distinct dry and wet seasons. Between seasons, a considerable shift in gut microbiota composition with cyclical features was displayed, with a number of taxa undetectable one season shown to reappear the next [76]. Succinivibrionaceae, Paraprevotellaceae, Spirochaetaceae, and Prevotellaceae families were among the most variable taxa across the seasons. These taxa, however, correspond to those that are often found to be rare or undetectable in Western populations [76]. Dietary fluctuations inform, at least in part, these cyclical variations though how other seasonal variants, including sun exposure, temperature, and relative time outdoors, impact the gut microbiota has yet to be explored [89]. In rodents, seasonal differences in the length of day has been shown to effect the composition of their microbial profile [90].

### 3.2. Capturing Long-Term Effect of Diet on the Gut Microbiota

Assessment of long-term diet–microbiome relationships have largely relied on epidemiological studies that tend to capture habitual diet through questionnaires such as food frequency questionnaires (FFQs). These studies have supported the role of habitual diet in shaping the microbial community, suggesting that through our habitual dietary choices, we are able to select substrates that provide a competitive environment for the gut microbiota. FFQs and comparable dietary questionnaires allow the assessment of the effect of diet on the gut microbiota through different approaches that include (i) the use of dietary indices to capture the overall dietary quality or type (e.g., Mediterranean diet or Western diet) dependent on quantities of nutrients or food groups consumed; (ii) the association between nutrients and phytochemicals with the gut microbiota; and (iii) the association between foods and food groups and the gut microbiota. 

#### 3.2.1. Dietary Diversity

A diverse diet, and in particular, the number of different types of plant foods consumed [35,78], has been associated with greater microbial alpha-diversity thought to provide an increased variety of substrates for numerous taxa proliferation [78,84]. In a 2019 observational longitudinal study by Johnson et al., daily 24-h food records and faecal shotgun metagenomes were collected from 34 participants for 17 days, from which they observed a positive relationship between dietary diversity and microbial stability [35]. The American Gut Project, a large citizen science open platform study, collected self-report dietary data (FFQs) and faecal samples amongst other measures from over 10,000 individuals, predominantly UK, USA, and Australian residents, confirmed these observations. Species Facealibacterium prausnitzii and genus Oscillospira, typically known to be SCFA fermenters, were increased in individuals who consumed more than 30 plant types a week in comparison to those who consumed less than 10 plant types [78]. Dietary diversity isn’t commonly accounted for within research methodologies and could explain why there can be variable outcomes when habitual diet is broadly investigated. For example, minimal differences have been shown between omnivores and vegetarians [35] although in some intervention studies, animal fat and protein predominant diets have been clearly associated with specific changes in gut microbial composition when compared to plant-based diets [67]. While counting the number of different plant foods reported can be a useful starting point, as displayed by The American Gut Project, incorporation of dietary diversity indices may provide further insight into the nuances of this diet diversity-microbial stability relationship such as the Healthy Food Diversity Index [91].

#### 3.2.2. Singular and Combined Nutrients

To describe the effect of dietary intake on the gut microbiota, studies tend to use a conventional nutrient profile approach derived from FFQs. Fibre is the most commonly accepted nutrient to exert a beneficial effect on microbiota composition, however other dietary components such as polyphenols, a group of anti-oxidants, are also thought to play an important role [92]. Polyphenols exert a dual effect on the gut microbiota as they can inhibit the growth of specific taxa, while enhancing the growth of others where they can be metabolised into bioavailable substrates for the host. A growing body of epidemiological studies have suggested that polyphenols, found in high levels in foods such as fruits, vegetables, cereals, coffee, tea and wine, are associated with a range of health promoting activities with a reduced risk of chronic diseases [92]. While a whole food approach acknowledges the symbiosis of nutrients with a matrix, singular nutrient and phytochemical observations in large longitudinal studies can still assist in building our understanding of diet–microbiota relations. Alongside fibre, magnesium, biotin, and vitamin E have all been shown to impact visceral fat mass accumulation mediated by the gut microbiota [79].

In contrary, the exclusion of an essential nutrients through dietary choices based on fad dietary advice, appropriate or inappropriate long use of therapeutic dietary strategies such as the low-FODMAP diet [93,94] and a gluten free diet may reduce microbial diversity [94]. In cystic fibrosis, bacteria generally considered markers of a healthy gut microbial profile such as Faecalibacterium, Roseburia, Akkermansia, and Bifidobacterium, were shown be decreased in a single-centre study on 43 individuals with cystic fibrosis [95]. Multiple factors for this loss of taxa were described by the authors, including diet. Those with cystic fibrosis are recommended to consume a diet high in protein and fat (and therefore a comparative reduction in carbohydrates) which has been previously associated with an increase in Firmicutes to Bacteriodetes ratio in mice [96], though dietary data was not collected within the cystic fibrosis study [95]. While diet has been shown to facilitate shifts in microbial composition in as little as three days, long-term elimination or excessive reduction of nutrients such as fermentable fibre produces microbial losses which are difficult to be reversed [84]. For example, the restriction fibre has been indirectly observed through Sanz’s investigation of the effect of a gluten-free diet on the microbiome of 10 individuals over the course of 30 days [66]. They found that quantities of beneficial bacteria decreased, in parallel with an increase of E. coli and total Enterobacteriaceae, bacteria typically associated with poor health thought to be in response to the reduced intake of polysaccharides (from 117 g to 63 g on average) [66]. Similarly, a Westernised diet is characterised by limited dietary diversity and a low fibre intake and has been greatly linked to an alteration of gut microbiota composition. The Western diet has been strongly associated with obesity and metabolic diseases, though the biological mechanisms remain unknown [97]. This may be as a result of increased levels of endotoxin-producing bacteria leading to metabolic endotoxemia. This state has often been attributed to a high-fat diet, though a mouse study demonstrated that microbial changes leading to this detrimental state were induced by a lack of fermentable fibre rather than dietary fat content [97,98].

#### 3.2.3. Dietary Indices

Associations between the gut microbiota and dietary indices, which describe food intake as a dietary pattern rather than individual food constituents, have also been explored. While these approaches assist in garnering an insight into the diet–microbiome relations they tend to simplify complex dietary intakes with detailed understanding of these interactions remaining elusive. Even so, dietary indices more closely represent an individual’s long-term habitual food intake in comparison to the observation of singular nutrients with consideration that foods are typically consumed in combination and not in isolation. A select number of studies have used dietary patterns to describe significant associations between food intake and measures of gut microbial composition. Bowyer et al. validated and compared three indices for their applicability to microbiome interventions, based on FFQ data from the TwinsUK cohort. The dietary index Healthy Eating Index (HEI) explained the most variance between diet and the microbiome across the three indices, with the strongest association with gut microbial composition than the other measures [91].

Shikany et al. used a data-driven factor analysis approach to assess dietary patterns without preconceived judgements of food groupings based on cultural or subjective opinions [80]. Adherence to a Western dietary pattern or a “prudent” pattern (based on high factor loadings of fruits, vegetables, nuts, fish, chicken and turkey without skin) on the gut microbiota of 517 older men were found to be associated with measures of beta-diversity, but not alpha-diversity [80]. Claesson et al. gathered FFQ data on 178 older men and women in Ireland at both community and long-term residential care facilities [81]. Four dietary groups including “low fat/high fibre”, “moderate fat/high fibre”, “moderate fat/low fibre” and “high fat/low fibre” were revealed by application of complete linkage clustering and Euclidean distances to the first eigenvector of their correspondence analysis. They found that dietary diversity was significantly associated with improved health parameters, with the grouping “low fat/high fibre” considered to have the most diverse diet and microbial profile, and “moderate fat/low fibre” and “high fat/low fibre” groupings the least [81]. Likewise, the Healthy Food Diversity index (HFD) was shown to be positively correlated with three microbiota diversity indices [81].

In children, two recent 2019 studies investigated the association of dietary patterns with the gut microbiota [99,100]. One cross-sectional study of 75 children between 2 to 9 years of age found that a number of food groups and nutrients were linked to differences in gut microbial composition consuming a Western dietary pattern, broadly defined by an increased consumption of refined carbohydrates, ultra-processed foods and high-fat animal products [100]. Diet was assessed through three 24-h dietary recalls, from which food groups were calculated. Food groups that were significantly associated with microbial structure (weighted unifrac distances) included plant protein, total fruit and total grain consumption [100]. Likewise, in a population of 4 to 8-year-old children (*n* = 22), Berding et al. found distinct differences in microbial composition for two habitual dietary patterns [99]. Faecal samples were taken at three timepoints over a 6-month period, with the participants habitual diet recorded for three days prior to each sample through 24-h records. Dietary patterns were then characterised through food group factor loadings. One dietary pattern, typified by consumption of fish, protein-rich foods, fruit juice and sweetened beverages, vegetables, fruits, snacks and sweets and kid’s meals, was associated with higher relative abundance of key bacterial taxa Bacteriodes, Prevotella and lower abundance of Bifidobacterium and displayed greater microbial stability over the 6-month period. A dietary pattern associated with grains, dairy, legumes, nuts and seeds, however, was shown to be associated with higher relative abundance of Cyanobacteria and Phascolarctobacterium and a lower abundance of Dorea and Eubacterium [99]. While it is generally accepted that habitual diet shapes the gut microbial composition and diversity in adulthood, these studies suggest that habitual diet also plays a role in pre-adolescence, or in fact, throughout the lifespan.

A limited number of studies investigate a change in microbial composition in response to long-term or habitual diet with further study of diet–microbiome relations required [6,75,76,77,78,79,80]. Dietary patterns that consider dietary quality and diversity may inform future habitual dietary strategies for durable microbial shifts, as opposed to a short-term transient initiative. For example, after 2 years, consumption of a Mediterranean dietary pattern and a low-fat dietary pattern has been shown to partially restore loss of keystone taxa in 33 participants with obesity and varying levels of metabolic dysfunction [101]. Regardless, contrary to most studies, habitual dietary intake should be taken into account in acute dietary interventions considering the long-lasting role dietary history has on the composition of the gut microbiota unless faced with an extreme dietary shift [102].

## 4. Limitations of Knowledge and Recommendations for the Future

Our understanding of the duration required for a dietary intervention to have an enduring impact on the gut microbiota, and consequently health, is hampered by several limitations. Firstly, there is a lack of long-term human studies, or indeed follow-ups of short-term dietary interventions, that seek to establish if a diet-induced modulation of the gut microbiota endures, though the wash-out periods of cross-sectional studies provide some indirect insight. Secondly, a number of studies have suggested that a host’s microbiota may be responsive or non-responsive to a dietary intervention based on the presence or absence of particular bacteria (e.g., fibre-degrading) within their core microbial population resulting in heterogeneous outcomes. An inter-generational decrease in fibre has been shown to lead to a reduction or extinction of these fibre-degrading bacteria in a mouse model [103]. A 2018 study of US first and second-generation immigrants were observed to have lost fibre-degrading bacterial enzymes that may have been associated with a reduction in dietary fibre consumption after migration from Southeast Asia [104]. The re-introduction of these lost species through the establishment of an ecological niche may be required to revert microbial diversity and richness to a higher state of ecological homeostasis. Thirdly, traditionally research has focused on the study of broad dietary indices or single and combined nutrients rather than foods within the context of specific dietary patterns. These approaches fail to acknowledge the synergistic effect within food matrixes. Capturing the complexity of diet itself remains a challenge that is not yet close to being overcome. This is mostly related to the high variability of dietary intake within and between individuals, leading to difficulty capturing and combining data usable for statistical modelling. Finally, the effect of transit time on the richness and composition of the gut microbiota is occasionally overlooked in current gut microbiome methodologies. While faecal richness is considered a hallmark of gut health, the composition of the faecal microbiota primarily reflects the stage of ecosystem development rather than communal stability [105]. For example, contrary to expectations a diet high in fermentable carbohydrates can lead to reduced microbial diversity within a faecal sample as a result of decreased transit time and softened stools [106]. As a result, collection of dietary data and its corresponding stool sample can be fraught with inconsistencies [107].

### 4.1. Increasing Emphasis on Habitual Diet Prior to Dietary Interventions and Analyses

Short-term dietary interventions frequently induce reproducible and profound shifts in gut microbial composition. However, these may draw a pre-emptory conclusion with limited dietary and faecal time-points investigating one dietary shift [108]. A number of studies have displayed that the gut microbiota typically reverts back to its baseline state post-intervention, with long-term diet thought to be a primary driver [109]. Establishing habitual dietary intakes and baseline gut microbiota composition as part of research methodologies could improve our understanding of the responsiveness of the gut microbiota to dietary interventions. While most studies have focused on the immediate effects of a dietary intervention, the long-term dietary history prior to the study initiation may provide further insight into the gut microbial profile and is as yet poorly understood [102].

Arguably, the long-term stability of the core species within a host’s gut microbiota is likely to be critical to associations with health and disease. The habitual diet of a host is considered a key driver in establishing this core microbial profile. While a 2019 mouse study by Yang et al. observed that these effects may vanish with the onset of extreme dietary conditions [102], it’s unknown if this is replicated in humans. Interindividual variability in response to diet is likely to be dependent on the baseline gut microbiota, and subsequently habitual diet. The level of microbial resilience can also be unique to a host’s gut microbiota, with some able to return to their original state after a perturbation, and others establishing a new, possibly pathological, profile [76]. Wu et al. introduced the concept of “permissive” and “restrictive” gut microbiotas, or “responders” and “non-responders”, as a possible explanation for differences in receptiveness to increased fibre intakes across various human populations. Wu et al. suggested that this may be due to an absence of certain key fibre-degrading species within the “restrictive” core gut microbiota, that were found to be present in the “permissive” core gut microbiota [110].

**Figure 2:**
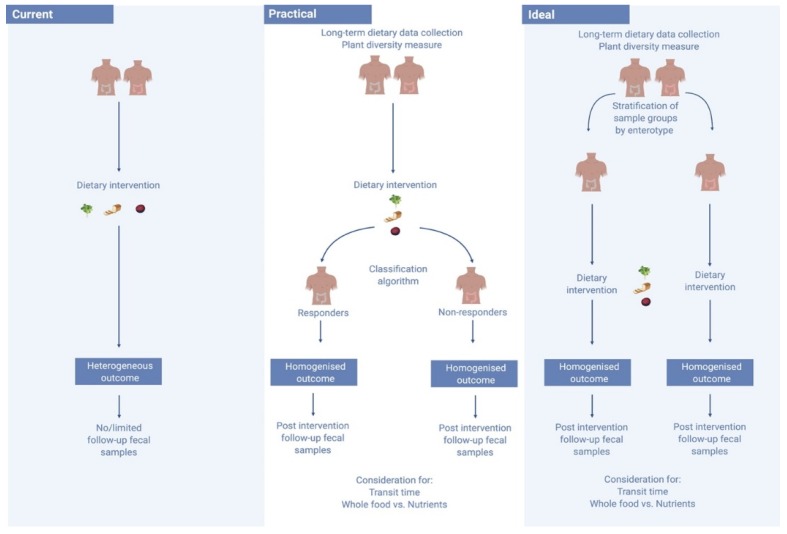
Moving from current to an ideal diet–microbiome study structure. Currently, diet–microbiome studies fail to consider a number of limitations, including the personalised microbiome, leading to heterogeneous outcomes. In an ideal setting, sample groups would be stratified by enterotype prior to the commencement of an intervention. Yet, faecal samples can take a lengthy time to process, stymieing study progress. A practical solution could be the use of a classification algorithm to stratify responders and non-responders with the hope of improving study outcomes. This image was generated using BioRender Software (http://www.biorender.com/).

Considering the context of the baseline gut environment may improve the predictability of the potential success of a dietary intervention. One 2015 study of 800 healthy subjects observed for 46,898 meals found large and at times conflicting inter-personal post-prandial responses to the dietary interventions [63]. The researchers found that by mapping an individual’s gut microbiota they were able to predict how fast their post-prandial response would be after eating a particular food - with each individual appearing to metabolise food very differently [63]. Non-responders could be characterised by high diversity, and individuals could be stratified into responders and non-responders based on the degree of microbiota stability [73]. Certain bacteria are also suggested to be more diet-responsive than others, with exposure in mice to these diet-responsive bacteria suggested to enhance the response to a dietary intervention [77]. Identification of Akkermansia muciniphila, for example, may predict the likelihood of success of an intervention [74]. A 2016 study by Dao et al. investigated the effects of a 6-week period of calorie restriction with a further 6 weeks of a weight stabilisation diet in 49 overweight and obese participants. The researchers evaluated the association between A. muciniphila abundance and gene richness of the host’s feacal sample, as well as diet and bio-clinical parameters [74]. Those with higher gene richness and a greater abundance of A. muciniphila displayed the healthiest metabolic status, particularly in regard to fasting plasma glucose, plasma triglycerides and distribution of body fat. Those with higher baseline of A. muciniphila also displayed greater amelioration in markers of insulin sensitivity after calorie restriction, and while these participants experienced a reduction in A. muciniphila abundance, it still remained significantly higher than in those with a lower baseline abundance [74]. As seen in Figure 2, defining and stratifying those with a diet-responsive gut microbiota within an intervention cohort may enhance the predictable performance of these dietary investigations, and would likely reduce the number of conflicting observations between similar studies [16]. In this way, identification of microbes associated with different dietary patterns can only be consistently supported by data which allows for the responsive profile of each individual’s gut microbiota.

### 4.2. Nutrient Centred Designs vs. Whole Food Approaches to Dietary Interventions

A usual human diet is composed of a variety of foods consumed throughout the day that all have unique nutrient makeup and matrix properties. The individual nutrients conventionally studied in research are, however, rarely consumed in isolation. The consumption of foods within a dietary pattern implies complex synergistic effects between and within multi-nutrient matrixes that may exert a greater microbial impact than one nutrient alone [111]. Recently, a study observing the consumption of red wine in three independent twin cohorts was found to be associated with increased alpha-diversity of the gut microbiota, yet, while not a nutrient, there was no association found with alcohol content itself [79]. Likewise, associations between nutrients that are commonly found in the same food sources make it difficult to examine their separate effects, confounded by components within a food’s composition that have not yet been identified [112]. A number of whole food controlled feeding studies have garnered an insight into the effect of specific foods on the gut microbial composition. A significant impact on the gut microbiota has previously been displayed through the consumption of cruciferous vegetables, walnuts, and almonds (consumption and processing) amongst others [113,114,115].

Experimental manipulation of macronutrient content invariably alters the dietary intake of other macronutrients. Food sources and overall dietary composition should ultimately be included in analysis of dietary intervention studies that aim to connect dietary changes to microbial compositional shifts. Incorporation of nutrient origin analysis using new multivariate methods as employed by Johnson et al. in their 2019 observational longitudinal study, or DNA metabarcoding techniques such as those employed by Reese et al., may provide further insight into the effect of food sources on the diet–microbiota relationship [35,116].

### 4.3. Symbiotics Provide an Opportunity to Selectively Alter Microbiome Composition

How do we ensure the engraftment of a specific bacterial strain? Perhaps the answer lies in the establishment of a metabolic niche that acts by promoting selected bacteria with targeted atypical dietary substrates. Synergistic symbiotic products combine prebiotics and probiotics to beneficially affect the host, and are developed to overcome possible survival difficulties for probiotics [117]. In this way, the prebiotic element is specifically designed to support the growth of the cognate probiotic. With the response to a dietary intervention being highly individual, synergistic symbiotics have the advantage of providing both the strain and its growth substrate in situ [47]. Using a mouse model Shepard et al. determined that through establishing a metabolic niche they were able to consistently promote a specific strain of bacteria irrespective of core microbial profile across all three gnotobiotic groups. The investigators selected and modified a unique bacterial strain that favours atypical nutrient substrates which when administered were able to give the strain a competitive advantage [48]. Importantly, the establishment of the strain was shown to be reversible once the nutrient substrate was removed from the diet. There is potential for new niches that could be created in conjunction with a customised diet enriched with specific nutrient substrates, and may promote desired strains consistently across individuals irrespective of core gut microbiota composition [48]. While this provides an interesting insight, research in humans is lacking, which may, in part, be related to the challenge of identifying a prebiotic that specifically and selectively enhances the probiotic strain of choice [47].

## 5. Conclusions

While we broadly understand the impact of diet on the gut microbiota, further insight into the effect and effect duration of specific dietary components remains elusive. A durable impact on the gut microbiota could allow a new state of ecological homeostasis to be reached, though likely consistent provision of nutritional substrates to the gut microbial environment are required for bacterial engraftment and proliferation. Currently, acute dietary interventions in humans have only observed transient microbial shifts in time periods of days to a number of weeks. Present knowledge of how dietary habits impact the gut microbiota in the long-term is limited by the lack of long-term dietary studies or indeed interventions with multiple faecal sample timepoints and post-intervention follow-ups, though indirectly, the washout periods of cross-over studies can provide some insight. Additionally, heterogeneity of research outcomes has impeded further insight into these diet–microbiota relations, likely due to personalised responses of the host microbiota. Stratifying study participants into “responders” and “non-responders” based on their baseline microbial profile may assist in eliciting improved outcomes. In future research, long-term dietary analysis should be integrated into acute diet interventions, with a need for further dietary data collected longitudinally to improve research results. Further long-term dietary interventions, including those that consider nutrient provenance, are required to investigate the potential for a durable diet-induced microbial shift. The wide range of individual microbial profiles should be acknowledged in order to explore personalised therapeutic strategies.

## Figures and Tables

**Table 1:** Short-term and Long-term Dietary Studies and the Gut Microbiota.

Primary Author	Year	Organism	Participants	Design	Dietary Data	Dietary Investigation	Length	Faecal Sample	Post-Intervention Faecal Sample	Change to Microbiome
**Short-term dietary interventions**										
C. Thiass [22]	2014	Mouse	10	Longitudinal	NA	Circadian rhythm: Ad libitum intake, for two light-dark cycles (12 h light, 12 h dark)	2 days	Every 6 h for two days	No	Significant (*p* < 0.05) diurnal fluctuations in the abundance of more than 15% of all bacterial operational taxonomic units (OTUs).
D. Zeevi [63]	2015	Humans	800	Longitudinal	FFQ, daily 24-h records	6898 habitual meals (total), and standardised meal per day	1 week	once	NA	People eating identical meals presented variability in post-meal blood glucose response.
G. Wu [6]	2011	Humans	10	Randomised, controlled feeding study	NA	Two treatment groups: (1) high-fat/low-fibre diet; (2) low-fat/high-fibre diet	10 days	10 days	No	Microbiome composition changed within 24 h of initiating a high-fat/low-fibre or low-fat/high-fibre diet, but enterotype remained stable throughout.
A. Johnson [35]	2019	Humans	34	Longitudinal	daily 24-h food records	Habitual diet	17 days	17 days	NA	Dietary diversity associates with microbiome stability. Daily dietary intake and microbiome composition are highly variable and personalised.
M. Ukhanova [64]	2014	Humans	18,16	Two controlled feeding, randomized, crossover studies (almond or pistachios)	NA	Three treatment groups: (1) no nuts; (2) 1.5 servings/d either almonds or pistachios; (3) 3 servings/d of either almonds or pistachios. 18-day intervention period. Inbetween: 2-week washout period.	18 days	6× samples: first and last day of each treatment period	No	Pistachio consumption had a greater impact on gut microbiota composition than almond consumption, including an increase in the number of potentially beneficial butyrate-producing bacteria. Pistachio consumption was associated with a decrease in the number of lactic acid bacteria.
L. David [18]	2013	Humans	11 (9 both diet arms)	Cross-over	Daily diet log with visual serving size portion guide, National Cancer Institute’s Diet History Questionnaire II (DHQ)	Two treatment groups: (1) Plant-based diet (5-days); (2) Animal-based diet (5-days). Prior to intervention: 4-days baseline habitual diet. Between: 6-days washout period. Post: 6-days washout period.	20 days	One sample a day: three baseline days and 2 days on each experimental diet selected.	Collected for 6 day washout period post each diet study arm	Short-term consumption of diets composed entirely of animal or plant products alters microbial community structure and overwhelms interindividual differences in microbial gene expression. However, microbiota composition returned to baseline within 3 days post intervention.
J. Karl [65]	2017	Humans	81	Randomized, controlled, parallel, controlled feeding	The Three-Factor Eating Questionnaire (weeks 2, 8), visual analog scales for hunger, satiety, prospective consumption, and diet satisfaction (weekly)	Two treatment groups: (1) whole grain diet; (2) refined grain diet. Prior to intervention: 2 week run in	6 weeks	All stools produced over 72 h during diet arm	No	Alpha-diversity and beta-diversity differed between groups at baseline (*P* < 0.05) but not after the intervention. Relative abundance of En- terobacteriaceae decreased and butyrate-producing Lachnospira and Roseburia increased in the WG compared with in the RG.
Y. Sanz [66]	2010	Humans	10	Preliminary	NA	Gluten-free diet (GFD)	30 days	Not reported	No	Numbers of beneficial bacteria decreased, while numbers of unhealthy bacteria increased parallel to reductions in the intake of polysaccharides after following the GFD.
F. De Filippis [67]	2015	Humans	153	Longitudinal	7-day food record	Three habitual diet groups: (1) Omnivore (*n* = 51); (2) vegetarian (*n* = 51); (3) vegan (*n* = 51)	3 weeks	3 samples: 1 per week	NA	High-level consumption of plant foods are associated with beneficial microbiome-related metabolomic profiles. Significant associations were detected between consumption of vegetable-based diets and increased levels of faecal short-chain fatty acids, Prevotella and Firmicutes.
J. Kaczmarek [68]	2019	Humans	18	controlled feeding, randomized, crossover study	NA	Treatment group: 200 g broccoli and 20 g daikon radish per day. Control: traditional American diet excluding all brassicas. Treatment/control period: 18 days; between: 24-day washout	60 days	3× samples: baseline, end of each treatment period	No	Broccoli consumption decreased the relative abundance of Firmicutes by 9% compared to control (*p* < 0.05), increased the relative abundance of Bacteroidetes by 10% compared to control (*p* < 0.03) and increased Bacteroides by 8% relative to control (*p* < 0.02).
S. Duncan [69]	2016	Humans	19	Experimental	NA	Two treatment groups: (1) high protein/medium carbohydrate (4weeks); (2) high protein/low carbohydrate (4 weeks). Prior to intervention: maintenance diet (3 days)	9 weeks	3× samples: 3 post first maintenance period, during last 2 days on each of the main diets	No	No significant change was seen in the relative counts of the Bacteroides, clostridial cluster XIVa, cluster IX, or cluster IV groups. In contrast, the *Roseburia* spp. and Eubacterium rectale subgroup of cluster XIVa and bifidobacteria decreased as carbohydrate intake decreased.
W. Russell [70]	2011	Humans	17	Randomised cross-over	NA	Two treatment groups: (1) high-protein and moderate-carbohydrate diet (HPMC) (4 weeks); (2) high-protein and low carbohydrate diet (HPLC) (4 weeks). Prior to intervention: weight maintenance diet (7 days)	9 weeks	3× samples: end of maintenance, HPMC and HPLC diet periods	No	The HPLC diet decreased the proportion of butyrate in faecal short-chain fatty acid concentrations, which was concomitant with a reduction in the Roseburia/Eubacterium rectale group of bacteria, and greatly reduced concentrations of fibre-derived, antioxidant phenolic acids such as ferulate and its derivatives.
A. Walker [19]	2010	Humans	14	Randomised cross-over design	NA	Two cross-over treatment groups: (1) Diet high in resistant starch (RS) (3 weeks); (2) Diet high in non-starch polysaccharides (NSP) (3 weeks); Prior to intervention: Initial maintenance diet protein/carbohydrate/fat% 13:52:35 and 27.7 g NSP (1 week). Post intervention: High protein, reduced carbohydrate weight loss (WL) diet (3 weeks).	10 weeks	Twice each week	No	Relatives of Ruminococcus bromii increased in most participants on the RS diet, accounting for a mean of 17% of total bacteria compared with 3.8% on the NSP diet, whereas the uncultured Oscillibacter group increased on the RS and WL diets. Relatives of Eubacterium rectale increased on RS (to mean 10.1%) but decreased, along with Collinsella aerofaciens, on WL.
E. Bellikci-koyu [71]	2019	Humans	22	Randomised parallel, controlled trial	24-h food recall × 2 (week 0, week 12)	Two treatment groups: (1) 180 mL/day kefir (12); (2) unfermented milk (10) control	12 weeks	2× samples: baseline, end of intervention	No	Kefir was associated with a significant increase in the relative abundance of Actinobacteria (*p* = 0.023) only. No significant change in the relative abundance of Bacteroidetes, Proteobacteria or Verrucomicrobia was obtained.
A. Cotillard [72]	2013	Humans	49	Control	7-day food record with interview by a dietitian	Intervention: energy-restricted high-protein diet (6 weeks). Control: weight-maintenance diet (6 weeks)	12 weeks	3× samples: baseline, 6 and 12 week	No	A significant increase of abundance of most gene clusters on energy restricted diet, however on weight-maintenance diet the abundance of 14 species decreased.
A. Salonen [73]	2014	Humans	14	Randomised cross-over design for RS and NSP interventions	NA	Two cross-over treatment groups: (1) Diet high in resistant starch (RS) (3 weeks); (2) Diet high in non-starch polysaccharides (NSP) (3 weeks); Prior to intervention: Initial maintenance diet protein/carbohydrate/fat% 13:52:35 and 27.7 g NSP (1 week). Post intervention: High protein, reduced carbohydrate weight loss (WL) diet (3 weeks).	10 weeks	4× samples: at end of each diet regimen	No	Multiple Ruminococcaceae phylotypes increased on the RS diet, whereas mostly Lachnospiraceae phylotypes increased on the NSP diet. Bifidobacteria decreased significantly on the WL diet. The RS diet decreased the diversity of the microbiota significantly. The dietary responsiveness of the individual’s microbiota varied substantially and associated inversely with its diversity.
M. Dao [74]	2016	Humans	49	Control	3× 7-day food records (prior to baseline, week 6 and week 12)	Intervention: calorie restricted diet (CR) enriched with fibre and protein (6 weeks). Control: weight stabilisation diet (6 weeks)	12 weeks	3× samples: baseline, week 6, week 12	No	Individuals with higher baseline A. muciniphila displayed greater improvement in insulin sensitivity markers and other clinical parameters after CR.
**Long-term dietary studies**										
H. Roager [75]	2014	Humans	62	Parallel randomised control trial	NA	Intervention: ad libitum New Nordic Diet (NND) (*n* = 36) (24–28 weeks). Control: ad libitum Average Danish Diet (ADD) (*n* = 26) (24–28 weeks). Prior to intervention: ADD (7–10 days)	6 months	2× samples: baseline, end of intervention	No	Negative association between Prevotella spp. and Bacteroides spp. did not reveal significant changes in 35 selected bacterial taxa resulting from the dietary interventions.
L. David [5]	2014	Humans	2	Longitudinal	daily 24-h food records	Habitual diet	1 year	Subject A: day 0–364. Subject B: day 0–252	NA	Human gut microbial landscapes are generally stable, but they can be quickly and profoundly altered.
S. Smits [76]	2017	Humans	188	Longitudinal	NA	Habitual hunter-gatherer diet of the Hadza tribe, Tanzania	>1year	350 samples	NA	Annual cyclic reconfiguration of the microbiome; some taxa became undetectable only to reappear in a subsequent season. Comparison of the Hadza data set with data collected from 18 populations in 16 countries reveals that gut community membership corresponds to modernization.
**Cross-sectional dietary studies**										
N. Griffin [77]	2016	Humans	170 (34 CRON, 198 AMER)	Cross-sectional	Food journals	Habitual dietary patterns (DP): (1) chronic calorie restriction with optimized intake of nutrients (CRON); (2) without prescribed or self-imposed dietary restrictions (AMER).	NA	1 sample	NA	AMER displayed less diverse faecal microbiota than those of individuals adhering to CRON.
D. McDonald [78]	2018	Humans	>10,000	Cross-sectional	FFQ, primary diet survey	Habitual diet	NA	1 sample	NA	The diversity of plants consumed are associated with microbial diversity with improved explanatory power vs. categorical variables (such as veganism).
C. Le Roy [79]	2019	Humans	916	Cross-sectional	FFQ	Beer, cider, red wine, white wine, spirits and total alcohol (sum)	NA	1 sample	NA	Red wine consumption was positively associated in a frequency dependent manner with alpha-diversity with even rare consumption displaying an effect
J. Shikany [80]	2019	Humans	517	Cross-sectional	FFQ	Habitual dietary patterns (DP) based on factor analysis: (Factor 1) ‘Western’ pattern (processed meats, refined grains, potatoes, eggs, sweets and salty snacks); (2) (Factor 2) ’prudent’ pattern (fruits, vegetables, nuts, fish, chicken and turkey without skin)	NA	1 sample	NA	Greater adherence to the Western pattern was positively associated with families Mogibacteriaceae and Veillonellaceae and genera Alistipes, Anaerotruncus, CC-115, Collinsella, Coprobacillus, Desulfovibrio, Dorea, Eubacterium, and Ruminococcus, while greater adherence to the prudent pattern was positively associated with order Streptophyta, family Victivallaceae, and genera Cetobacterium, Clostridium, Faecalibacterium, Lachnospira, Paraprevotella, and Veillonella
M. Claesson [81]	2012	Humans	178	Cross-sectional	FFQ	Habitual dietary patterns (DP): (1) low fat/high fibre; (2) moderate fat/high fibre; (3) moderate fat/low fibre; (4) high fat/low fibre. Residential location was also considered (community vs. long-term care homes)	Na	1 sample	NA	The healthy food diversity index (HFD23) positively correlated with three microbiota diversity indices and all four indices showed significant differences between community and long-stay subjects indicating that a healthy, diverse diet promotes a more diverse gut microbiota.
G. Wu [6]	2011	Humans	98	Cross-sectional	FFQ × 1, 24-h food record × 3	Habitual diet	NA	1 sample	NA	72 and 97 microbiome-associated nutrients were identified in 24-h recall and FFQ. Long-term diet was correlated with enterotype clustering.

Acronyms: Not Applicable (NA); Food Frequency Questionnaire (FFQ); Dietary pattern (DP).
